# The Antioxidant and Anti-Inflammatory Effects of the Main Carotenoids from Tomatoes via Nrf2 and NF-κB Signaling Pathways

**DOI:** 10.3390/nu15214652

**Published:** 2023-11-02

**Authors:** Wenxiu Ba, Wenzhen Xu, Zeyuan Deng, Bing Zhang, Liufeng Zheng, Hongyan Li

**Affiliations:** 1 State Key Laboratory of Food Science and Resources, Nanchang University, Nanchang 330031, China; 407900210017@email.ncu.edu.cn (W.B.); xuwz66@outlook.com (W.X.); dengzy@ncu.edu.cn (Z.D.); zhangbingair@hotmail.com (B.Z.); zhenglf2018@ncu.edu.cn (L.Z.); 2 International Institute of Food Innovation, Nanchang University, Nanchang 330051, China

**Keywords:** carotenoids, Nrf2 signaling pathway, NF-κB signaling pathway, anti-inflammatory effect, antioxidant effect

## Abstract

Oxidative stress and inflammation are crucial factors in the development of cardiovascular diseases. In previous research, the oxidative stress and inflammation models have frequently been explored independently. In the current study, we investigated the antioxidant and anti-inflammatory effects of tomato extract and its two main carotenoids (lutein and lycopene) with various concentrations using a rat cardiomyocyte model of co-existing oxidative stress and persistent chronic inflammation. It was discovered that the antioxidant effects of 0.5–5 μM lutein, 0.5–5 μM lycopene, and 50–200 μg/mL tomato extract increased in a dose-dependent manner. However, the pro-oxidation effects emerged by measuring the antioxidant-related indices, including the levels of ROS, SOD, and GPX in H9c2 cells as concentrations exceeded those mentioned above. The anti-inflammatory effects of lutein, lycopene, and tomato extract were simultaneously strengthened with higher concentrations, potentially due to the suppression of the NF-κB signaling pathway. Furthermore, high concentrations of lutein, lycopene, and tomato extract potentially regulated Nrf2/HO-1 and NF-κB signaling pathways dependent on TGF-1β and IL-10 to demonstrate high concentrations of pro-oxidation and anti-inflammation effects. Our findings indicate that the dose–effect regulatory mechanisms of antioxidant and anti-inflammatory properties among lutein, lycopene, and tomato extract will be advantageous in developing more effective therapeutic strategies to prevent cardiovascular diseases.

## 1. Introduction

Numerous epidemiological studies have demonstrated that cardiovascular disease (CVD) is among the significant causes of mortality in humans [1]. The occurrence of oxidative stress and inflammation can increase the risk of cardiovascular disease as well as other associated conditions. Oxidative stress is a noticeable feature of CVD, which includes atherosclerosis and myocardial infarction [2,3]. Simultaneously, the initial stage of CVD is characterized by the increase in the acute phase proteins and inflammatory mediators, and the inflammatory response persists throughout the entire stage of CVD [4,5].

Specific correlations exist between oxidative stress and inflammation during disease development. For instance, the occurrence of oxidative stress and an inflammatory response would both impede the insulin response and increase the risk of cardiovascular disease and other related illnesses [6]. Likewise, chronic inflammation is typically associated with an increased risk of cancer due to the enormous amount of oxidative DNA damage, the detrimental effects of inflammation-induced reactive oxygen and nitrogen species (RONS) on DNA repair enzymes, and the resulting rise in mutation rate [7]. Meanwhile, there are special interactions between pathways associated with oxidative stress and inflammatory damage in pathological processes. Phytochemicals have been implicated in the development of oxidative stress and chronic inflammation [8]. For example, propolis flavonoids may activate the Nrf2 pathway and inhibit the NF-κB pathway to maintain the balance of antioxidant and anti-inflammatory effects [9].

Abundance investigations have demonstrated that tomatoes possess antioxidation [10], anti-inflammatory [11], hypoglycemic [12,13], light-protection [14], anti-angiogenesis [15], anti-virus [16], and other biological activities. Carotenoids from tomatoes are enriched in lutein and lycopene, which are significant reasons for particular antioxidant and anti-inflammatory effects. Increasing evidence indicates that lutein shows prominent protective effects on CVD [17]. Lutein can maintain the balance of heart metabolism by preventing or reducing tissue oxidation [18]. In addition, lutein supplementation notably reduces the serum levels of inflammatory cytokines and low density lipoprotein during the pathological process of atherosclerosis [19]. Lycopene, which contains eleven conjugated double bonds, is the main and most powerful carotenoid in human blood [20]. Lycopene holds a balanced state of oxidation and reduction by reducing the production of ROS and ONOO^−^, thus preventing the occurrence of CVD [21]. Additionally, lycopene is capable of inhibiting lipopolysaccharide (LPS) to induce NF-κB activation, moderating the expression of cell adhesion factors, moderating vascular permeability, and inhibiting the deterioration of vascular inflammation [22].

It is noteworthy that there are corresponding dose–effect relationships between the antioxidant and anti-inflammatory effects of carotenoids. Clinical studies confirmed that the oral administration of lutein at a higher concentration (0.42 μmol/L) in patients with atherosclerosis compared with the low lutein group (0.15 μmol/L) decreased the aortic wall thickness by 80% [23]. Moreover, 20 mg/kg and 60 mg/kg of lycopene enhanced the glucose and lipid metabolism, and reduced the level of inflammatory factors in rats, for which the impact of 60 mg/kg was more significant [24]. However, the existing related investigations into the dose–effect relationships between the antioxidant and anti-inflammatory functions of tomato and its main carotenoids (lutein and lycopene) are not comprehensive enough.

H9c2 cells are derived from clonal cell lines of BD1X rat embryonic heart tissue [25].The resulting cloned cell lines are particularly useful for biochemical analysis because they are more phenotypically homogeneous than whole tissue or primary cultures. The H9c2 cells were selected to have more similarity to the oxidative damage and chronic inflammation in cardiac muscle cells in the development of cardiovascular diseases. Conventional cardiomyocyte injury models only involve inducing oxidative stress using H_2_O_2_ [26] or LPS, or inflammatory damage with high concentrations of TNF-α or LPS [27]. In our previous research, rat cardiomyocytes were co-induced with low concentrations of TNF-α and H_2_O_2_ in order to mimic the simultaneous effects of oxidative damage and chronic inflammation. The model was validated using flow cytometry, Western blot, and Q-PCR. A significant increase in intracellular ROS, an increase in MDA in oxidative stress indicators, and a decrease in the antioxidant enzyme SOD and the expression of inflammatory factor proteins and mRNA downstream of the NF-κB signaling pathway were all found to occur in rat cardiomyocytes after 200 μM H_2_O_2_ and 80 ng/mL TNF-α co-induced the cells [28]. For the purpose of testing anti-inflammatory medications and treating inflammatory illnesses, the establishment of an oxidative stress–chronic inflammation model is crucial.

Therefore, this study took full advantage of the established oxidative stress injury and inflammation of chronic diseases of the rat myocardial cell model to explore the effects of different concentrations of lutein, lycopene, and tomato extract on the level of ROS and the expression of pro-inflammatory and anti-inflammatory factors and related antioxidant enzymes. By exploring the expression of proteins related to the Nrf2 and NF-κB pathways, this work investigated the dose–effect mechanism of the regulation of antioxidant and anti-inflammatory functions among lutein, lycopene, and tomato extract. The present study provides guidance for research into the dose effect of carotenoids and provides a theoretical basis for the prevention of chronic metabolic diseases such as CVD.

## 2. Materials and Methods

### 2.1. Materials

H9c2 cells were purchased from Procell Life Science & Technology Co., Ltd. (Wuhan, China). Fetal bovine serum (FBS) and Dulbecco’s modified Eagle’s medium (DMEM) were purchased from Biological Industries (Shanghai, China). Tris-buffered saline-Tween-20 (TBST), penicillin-streptomycin liquid, lutein standards, and lycopene standards were purchased from Solarbio life sciences (Beijing, China). Tetrahydrofuran, acetone, ethanol, dimethyl sulfoxide (DMSO), and 30% hydrogen peroxide (H_2_O_2_) were purchased from Damao Co., Ltd. (Tianjin, China). Reactive Oxygen Species Assay Kit, Cell Counting Kit-8, Catalase Assay Kit, Total Superoxide Dismutase Assay Kit with WST-8, Nitric Oxide Synthase Assay Kit, and Griess Reagent were purchased from Beyotime Biotechnology (Shanghai, China). Nrf2 (1:1000, AB_881705), ICAM (1:5000, AB_2814769), VCAM (1:3000, AB_2721053), HO-1 (1:3000, AB_11156457), and NQO1 (1:3000, AB_1603750) were purchased from Abcam (Cambridge, UK). Phospho-NF-κB p65 (1:1000, 3033) and IL-6 (1:1000, AB_2889391) were purchased from Cell Signaling Technology (Danvers, MA, USA). β-actin (1:3000, AB_2941082) was purchased from Santa Cruz (Dallas, TX, USA). Horseradish peroxidase-conjugated anti-rabbit (1:3000, 21804) and anti-mouse (1:3000, 21816) were purchased from Signalway Antibody (Nanjing, China). PrimeScriptTM RT Reagent Kit with Gdna Eraser (Perfect Real Time) and Beastar^®^ qPCR mastermix (SYBR Green) were purchased from TaKaRa Bio (Shiga, Japan).

### 2.2. Sample Preparation

The lyophilized tomatoes were crushed into powder, and the pre-treated tomato powder was obtained through a 100-mesh sieve. The pre-treated tomato powder was soaked in acetone-ethanol solution (1:1) at a ratio of 1:20 (*w*/*v*), vortexed, and then sonicated for 30 min at the power of 100 W and 40 kHz. Immediately, it was stirred at a speed of 150 rpm/min for 4 h and centrifuged at 4200 rpm for 5 min. The supernatant was gathered after repeating the previous procedure three times [29]. Finally, the extract was blown dry with nitrogen to obtain the tomato extract powder, which was weighed, redissolved in tetrahydrofuran, and kept at −80 °C. The suspension of tomato extract containing 10 mg/mL was kept for subsequent cell tests. Strictly light-proof conditions were used for all operations.

### 2.3. HPLC

A total of 1 mL diluted tomato extract was accurately aspirated after re-dissolution, filtered through a 0.22 μm microporous membrane, and left for measurement. The extracted carotenoids and lycopene and lutein standards were separated on an Agilent 1260 series high-performance liquid chromatograph with the following HPLC separation conditions: a Kinetex XB-C18 100A liquid chromatographic column with mobile phases of 80% methanol (A) and acetonitrile-tetrahydrofuran (90:10) (B), a column temperature of 30 °C, a DAD detection wavelength of 450 nm, and injection volume of 5 μL. The gradient protocol was: 0–2 min, 40–60% B; 3–10 min, 60–100% B; 11–15 min, 100% B; 16–25 min, 100–60% B; 26–40 min, 60–40% B, with a flow rate of 0.5 mL/min and a post-run time of 2 min [30].

Lutein standard stock solution and lycopene standard stock solution were prepared by dissolving approximately 1 mg of accurately weighed lutein standard or lycopene standard in acetone solution. Lutein and lycopene working solutions were prepared by dilution of standard stock solution with acetone to concentrations of 0.2, 0.4, 0.6, 0.8, and 1 mg/mL. The injection volume was 5 µL to produce the standard curve graph.

### 2.4. Cell Culture and Treatment

H9c2 cells were routinely incubated in a CO_2_ incubator at 37 °C and 5% CO_2_. H9c2 cells were proliferated in DMEM augmented with 10% FBS, 1% sodium pyruvate, and 1% penicillin and streptomycin. Before undergoing various treatments, H9c2 cells were seeded on the appropriate plates at a density of 5 × 10^4^ cells/mL. Lutein, lycopene, and tomato extract were dissolved in DMSO and stored at −80 °C, and diluted in DMEM in different proportions before cell incubation [30].

### 2.5. Cell Viability

H9c2 cells were seeded into 96-well plates. As the cell covering density reached 70–90%, the cells were then treated for 12 h with various treatments. The different treatment groups included the control group, 200 μM H_2_O_2_ combined with 80 ng/mL TNF-α model group, and treatment groups with various concentrations of lutein, lycopene, and tomato extract. After that, 10 μL of CCK-8 solution and 90 μL of DMEM were accurately transferred to each pore. Cells were incubated in standard culture conditions for 1 h, and the optical density (OD) value was measured at 450 nm of the EXL800 automatic microplate reader (Biotek, Winooski, VT, USA).

### 2.6. Production of Reactive Oxygen Species (ROS)

#### 2.6.1. Confocal Laser Method

A total of 1 mL of H9c2 cells suspension was seeded into a small vessel of a confocal laser. Afterward, when the coverage density of cells reached 60–70%, the cells were subjected to various treatments. The different treated groups included the control group, the 200 μM H_2_O_2_ combined with 80 ng/mL TNF-α model group, and treatment groups containing different concentrations of lutein, lycopene, and tomato extract. Three parallel treatments were set up for each group. DCFH-DA was prepared in 5 μmol/L by serum-free medium, and 500 μL DCFH-DA was added to each well and incubated in a 37 °C incubator for 20 min. The small vessel was placed under a Confocal Laser Scanning Microscope (Leica TCS SP8, Leica Microsystems GmbH, Wetzlar, Germany) for observation, and pictures were taken using the excitation wavelength of 488 nm and the emission wavelength of 525 nm.

#### 2.6.2. Production of Reactive Oxygen Species (ROS)

Intracellular ROS levels were ascertained through flow cytometry (BD FACS, Becton Dickinson Co., New York, NY, USA) with a fluorescent probe DCF-DA. Briefly, H9c2 cells were seeded inside a twelve-well plate. When the coverage density of cells reached 60–70%, the cells were subjected to various treatments for 12 h. DCFH-DA was prepared in 5 μmol/L using the serum-free medium, then cleaned three times by PBS. A quantity of 500 μL DCFH-DA was added to each well and incubated in a 37 °C incubator for 20 min, and then washed three times with serum-free medium. Furthermore, 0.15% trypsin digestion solution was added, and cells were collected and centrifuged (1500 rpm, 4 °C, 5 min), and the fluorescence intensity was ascertained using flow cytometry (BD FACS, Becton Dickinson Co., Franklin Lakes, NJ, USA).

### 2.7. SOD and GPX Assays

H9c2 cells (5 × 10^4^ cells per well) were seeded inside a twelve-well plate. When the coverage density of cells reached 70–90%, the original medium was discarded, and the cells were subjected to various treatments for 12 h. The different treated groups included the control group, the 200 μM H_2_O_2_ combined with 80 ng/mL TNF-α model group, and treatment groups containing different concentrations of lutein, lycopene, and tomato extract.

After lysis using a tissue lysis solution, the supernatant of H9c2 cell was gathered. After that, 50 µL of the cell supernatant was added to the corresponding 96-well plate. The intracellular SOD activity was measured using the Total Superoxide Dismutase Assay Kit. Then, a EXL800 automatic microplate reader (Biotek, Winooski, VT, USA) was used to measure the absorbance at 560 nm.

Likewise, after lysis using a tissue lysis solution, the supernatant of H9c2 cells was gathered. After that, 50 µL of the cell supernatant was added to the corresponding 96-well plate. The intracellular GPX activity was measured using the GPX Assay Kit. The organic peroxide reagent provided in this kit is cumene hydroperoxide. Therefore, the activity of glutathione peroxidase can be more specifically detected. Then, using a EXL800 automatic microplate reader (Biotek, Winooski, VT, USA), the absorbance at 340 nm was determined.

### 2.8. iNOS Assays

H9c2 cells (5 × 10^4^ cells per well) were seeded inside a six-well plate when the coverage density of cells reached 60–70%. Moreover, the cells were subjected to various treatments for 24 h. The different treated groups included the control group, the 200 μM H_2_O_2_ combined with 80 ng/mL TNF-α model group, and treatment groups containing different concentrations of lutein, lycopene, and tomato extract. After lysis using a tissue lysis solution, the supernatant of the H9c2 cell was gathered. After that, 200 µL of the cell supernatant was added to the corresponding 96-well plate, various drugs were successively added, and it was incubated for 24 h. A quantity of 100 μL of buffer was added, an additional 100 μL of Assay Reaction Solution was added, and it was gently mixed. Finally, the absorbance at 515 nm was measured in an EXL800 automatic microplate reader (Biotek, Winooski, VT, USA).

### 2.9. Extraction of the Whole-Cell Protein, Cytosolic Protein, and Nuclear Protein

H9c2 cells (5 × 10^4^ cells per well) were seeded inside a six-well plate when the coverage density of cells reached 70–90%. Cells were washed with PBS three times following various treatments. Cells were added to the prepared cold lysate (10 μL protein phosphatase inhibitor and 10 μL benzoyl sulfonyl fluoride were separately prepared in 1 mL RAPA) and cleaved on ice for 30 min. Whole-cell proteins were isolated using a protein extraction kit (Shanghai, China). The supernatant was then collected as whole-cell proteins. According to the manufacturer’s instructions, cytosolic and nuclear proteins were isolated using a nuclear and cytoplasmic extraction kit from Beyotime Biotechnology. The samples were stored at −80 °C and the protein content was determined using a BCA Kit (Beyotime Biotechnology, Shanghai, China).

### 2.10. siRNA Interference Technique Silencing the Expression of TGF-β1/IL-10 Gene

H9c2 cells were seeded in 12-well cell culture plates with 0.5 mL of medium per well a day prior to transfection. Transfection started once the cell growth reached 60–70% and the siRNA-liposome complex was prepared as described below. Liquid A (50 μL of Opti-MEM medium combined with 2 μL of siRNA) and liquid B (50 μL of Opti-MEM medium combined with 1 μL of lipo2000) were carefully combined, and kept for 20 min. The cells were washed with Opti-MEM medium once, and 400 μL of Opti-MEM medium was added to each well. The SirNa-liposome complex was then added and distributed evenly for 4–6 h. After 10% DMEM cell medium with FBS was substituted, the process was continued in the incubator for another 12 h.

### 2.11. The Gene Expression Levels of Nrf2 Pathway and NF-κB Pathway after Gene Silencing Were Determined by Q-PCR

Following transfection, the cells were exposed to lutein, lycopene, and tomato extract at various concentrations for 12 h, and the oxidative and inflammatory damage was induced by combining TNF-α and H_2_O_2_. Total RNA in H9c2 cells of each group was extracted. In order to determine the target mRNA expression of each sample, a reverse transcription kit was applied for reverse transcription, and SYBR Green PCR Master Mix was used to amplify the products after reverse transcription. Finally, a PCR instrument was used to amplify the products after reverse transcription. The primer sequence for the gene Nrf2, NF-κB, and HO-1, which was discovered during the quantitative fluorescence PCR stage, is provided in Table S1 of the Supporting Information.

### 2.12. Western Blot Analysis

Proteins were combined with sodium dodecyl sulfate (SDS) loading buffer. Proteins were resolved and identified using 10% sodium dodecyl sulfate–polyacrylamide gel electrophoresis (SDS-PAGE) and transferred to PVDF membranes using a continuous current. PVDF membranes were incubated with the matching primary antibody for 2 h at room temperature (about 25 °C) after being blocked with 5% skimmed milk in Tris-buffered saline-Tween-20. PVDF membranes were incubated with the corresponding primary against VCAM (1:3000, AB_2721053), ICAM (1:5000, AB_2814769), IL-6 (1:1000, AB_2889391), TNF-α (1:1000, AB_2935774); Nrf2 (1:1000, AB_881705), HO-1 (1:3000, AB_11156457), phospho-NF-κB p65 (1:1000, 3033), and β-actin (1:3000, AB_2941082) overnight at 4 °C. After washing with TBST, the membranes were incubated for 2 h at room temperature with horseradish peroxidase-conjugated anti-mouse (1:3000, 21816) or anti-rabbit (1:3000, 21804) secondary antibodies. After three TBST washes, target bands were finally visible in enhanced chemiluminescence (ECL) detection solution using the ECL technique (Image LabTM Touch Software v3.0.1, Bio-Rad, Hercules, CA, USA). An imaging analyzer was used to measure the intensity of target bands (Image Lab, National Institutes of Health, USA). Histone H3 or -actin was used to normalize the relative expression of proteins.

### 2.13. Statistical Analysis

The data were statistically processed using GraphPad Prism 8.0 software (San Diego, CA, USA). SPSS 26.0 univariate analysis of variance was used for statistical analysis, and all data are represented by mean ± standard error (mean ± SEM). Duncan’s multiple comparison was used for significance analysis, where *p* < 0.05 indicated a statistically significant difference.

## 3. Results

### 3.1. Extraction and Identification of the Major Carotenoids from Tomatoes

The major carotenoids in tomato extract were identified by comparing their retention times and absorption spectra with lutein standards and lycopene standards. The major carotenoids in tomatoes were lutein (peak 1) and lycopene (peak 4) upon comparison with lutein standards (Figure S1A,B) [31]. After checking the literature, peak 2 may be the isomer of lutein, peaks 3 and 5 may be the isomers of lycopene, and peak 6 was β-carotene based on their retention times and response values (Figure S1C) [32].

### 3.2. Effects of Lutein, Lycopene, and Tomato Extract on the Viability of H9c2 Cells

Treatment with different concentrations of lutein (1–100 μM), lycopene (0.1–100 μM), and tomato extract (1–1000 μg/mL) reduced the cell viability in a dose-dependent manner (Figure S2). The cell viability of H9c2 cells was less than 95% at doses higher than 20 μM for lutein, 20 μM for lycopene, and 400 μg/mL for tomato extract, indicating a significant decrease in cell viability above these concentrations (*p* < 0.05). In this experiment, the concentration dose was determined with a cell survival rate higher than 95%. Therefore, lutein below 20 μM, lycopene below 20 μM, and tomato extract below 400 μg/mL were used for the subsequent experiments (Figure S2).

### 3.3. Effects of Lutein, Lycopene, and Tomato Extract on ROS Levels in H9c2 Cells

#### 3.3.1. Confocal Laser Method for the Intracellular ROS Detection

**Figure 1:**
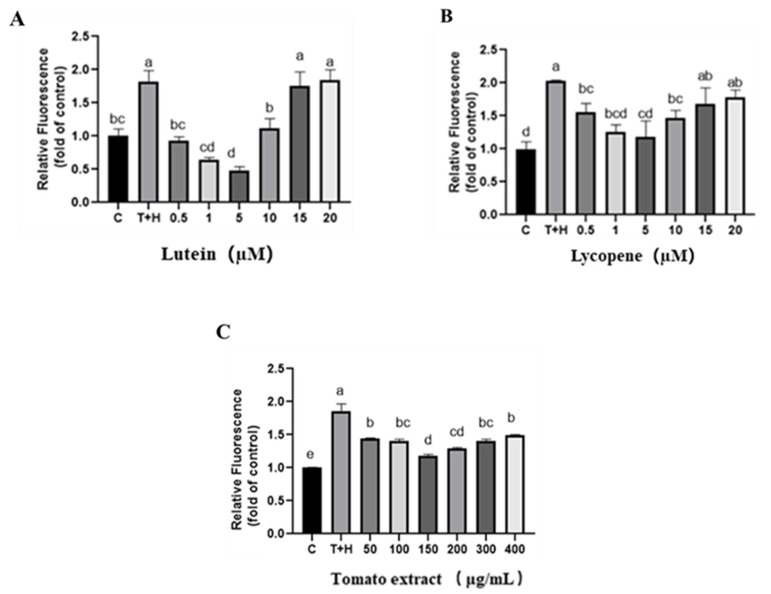
ROS levels induced by lutein, lycopene, and tomato extract in 200 μM H_2_O_2_ and 80 ng/mL TNF-α co-induced H9c2 cells and measured using the confocal laser method. (**A**) ROS levels of H9c2 cells treated with varying concentrations of lutein. (**B**) ROS levels of H9c2 cells treated with varying concentrations of lycopene. (**C**) ROS levels of H9c2 cells treated with varying concentrations of tomato extract. The horizontal coordinate C represents control group, and the horizontal coordinate T + H represents 200 μM H_2_O_2_ combined with 80 ng/mL TNF-α model group. Different letters (a, b, c, d, e) indicate significant differences between groups (*p* < 0.05).

ROS such as oxygen radicals and peroxides are by-products of normal mitochondrial and cellular oxygen metabolism. High levels of ROS can disrupt cellular homeostasis, and the excessive apoptosis caused by increased intracellular ROS production may induce organ dysfunction [33]. Therefore, the present study initially examined the changes in ROS levels induced by different concentrations of lutein, lycopene, and tomato extract using confocal laser microscopy. ROS levels were significantly higher in the T + H group (80 ng/mL TNF-α + 200 µM H_2_O_2_) compared to the control group (*p* < 0.05). Pretreatment with different doses of lutein, lycopene, and tomato extract improved the oxidative stress induced by 80 ng/mL TNF-α + 200 µM H_2_O_2_ to different degrees (*p* < 0.05). ROS levels decreased continuously and in a dose-dependent manner when the concentrations of lutein, lycopene, and tomato extract were 0.5–5 μM, 0.5–5 μM, and 50–150 μg/mL, respectively. Instead, as the concentrations of lutein (10–20 μM), lycopene (10–20 μM), and tomato extract (200–400 μg/mL) increased, the ROS levels also increased gradually and in a dose-dependent manner (Figure 1).

#### 3.3.2. Detection of the Intracellular ROS by Flow Cytometry

**Figure 2:**
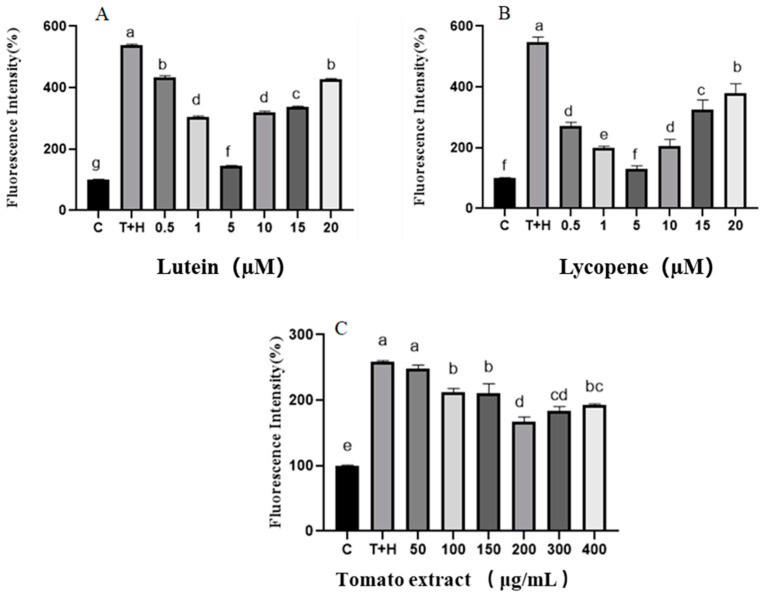
ROS levels induced by lutein, lycopene, and tomato extract in 200 μM H_2_O_2_ and 80 ng/mL TNF-α co-induced H9c2 cells and measured by flow cytometry. (**A**) ROS levels of H9c2 cells treated with varying concentrations of lutein. (**B**) ROS levels of H9c2 cells treated with varying concentrations of lycopene. (**C**) ROS levels of H9c2 cells treated with varying concentrations of tomato extract. The horizontal coordinate C represents control group and the horizontal coordinate T + H represents 200 μM H_2_O_2_ combined with 80 ng/mL TNF-α model group. Different letters (a, b, c, d, e, f, g) indicate significant differences between groups (*p* < 0.05).

The ROS levels measured via confocal laser microscopy were only a semi-quantitative assay; thus, flow cytometry was also used to qualitatively and quantitatively verify the intracellular ROS levels in different concentrations of lutein, lycopene, and tomato extract treatment groups. The results obtained by flow cytometry were consistent with those from confocal laser microscopy (Figure 2).

### 3.4. Effect of Lutein, Lycopene, and Tomato Extract on the Antioxidant Enzymes in H9c2 Cells

#### 3.4.1. SOD

**Figure 3:**
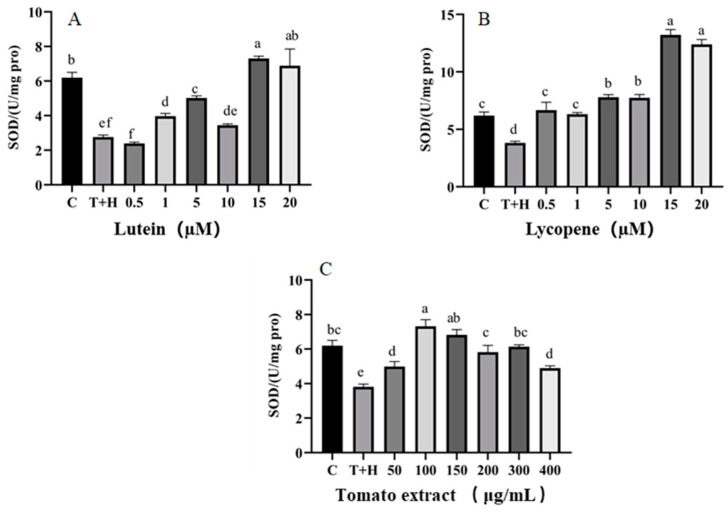
SOD levels induced by lutein, lycopene, and tomato extract in 200 μM H_2_O_2_ and 80 ng/mL TNF-α co-induced H9c2 cells. (**A**) SOD levels of H9c2 cells treated with varying concentrations of lutein. (**B**) SOD levels of H9c2 cells treated with varying concentrations of lycopene. (**C**) SOD levels of H9c2 cells treated with varying concentrations of tomato extract. Different letters (a, b, c, d, e, f) indicate significant differences between groups (*p* < 0.05).

SOD plays a significant role in antioxidative stress in that superoxide free radicals are scavenged to maintain cellular homeostasis [34]. SOD activity was higher in the groups treated with 0.5–20 μM lutein, 0.5–20 μM lycopene, and 50–400 μg/mL tomato extract compared to the T + H group (80 ng/mL TNF-α+ 200 μM H_2_O_2_, 3.81 ± 0.15 U/mg protein). In a dose-dependent manner, 50–150 μg/mL of tomato extract, 0.5–5 μM lutein, and 0.5–10 μM lycopene increased activity of SOD (Figure 3). The results suggest that a pro-oxidant effect was found at high concentrations of lutein (15–20 μM), lycopene (15–20 μM), and tomato extract (200–400 μg/mL). For example, when the concentration of tomato extract was increased from 50 to 150 μg/mL, the SOD enzyme activity increased from 4.99 ± 0.28 U/mg protein to 7.32 ± 0.38 U/mg protein, and when the concentration of tomato extract continued to increase to 400 μg/mL, the SOD enzyme activity decreased to 4.9 ± 0.12 U/mg protein.

#### 3.4.2. GPX

**Figure 4:**
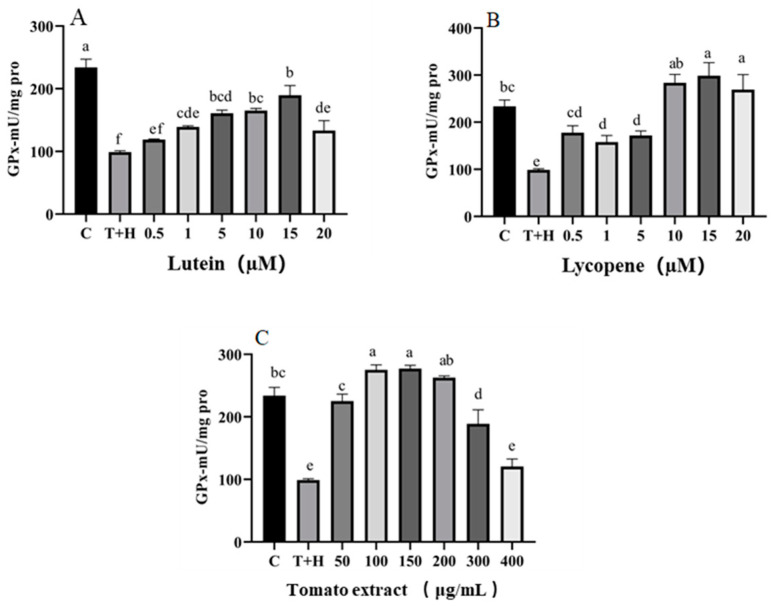
GPX levels induced by lutein, lycopene, and tomato extract in 200 μM H_2_O_2_ and 80 ng/mL TNF-α co-induced H9c2 cells. (**A**) GPX levels of H9c2 cells treated with varying concentrations of lutein. (**B**) GPX levels of H9c2 cells treated with varying concentrations of lycopene. (**C**) GPX levels of H9c2 cells treated with varying concentrations of tomato extract. Different letters (a, b, c, d, e, f) indicate significant differences between groups (*p* < 0.05).

The T + H group (80 ng/mL TNF-α+ 200 μM H_2_O_2_, 98.86 U/mg protein) showed significantly lower GPX activity compared to the control group (233.84 U/mg protein) (*p* < 0.05); the groups treated with 0.5–20 μM lutein, 0.5–20 μM lycopene, and 50–400 μg/mL tomato extract all exhibited higher GPX activity compared to the T + H group. Moreover, GPX levels decreased after pretreatment with 15–20 μM lutein, 10–20 μM lycopene, and 200–400 μg/mL tomato extract, suggesting a pro-oxidation effect (Figure 4).

### 3.5. Effects of Lutein, Lycopene, and Tomato Extract on the Expression of Pro-Inflammatory Cytokines in H9c2 Cells

**Figure 5:**
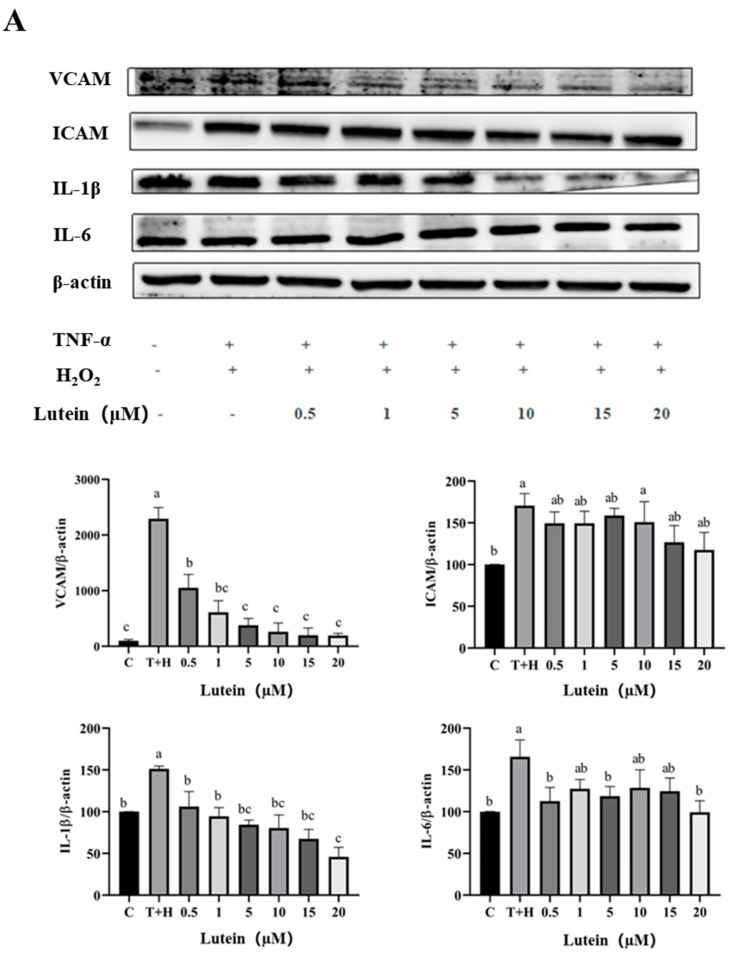

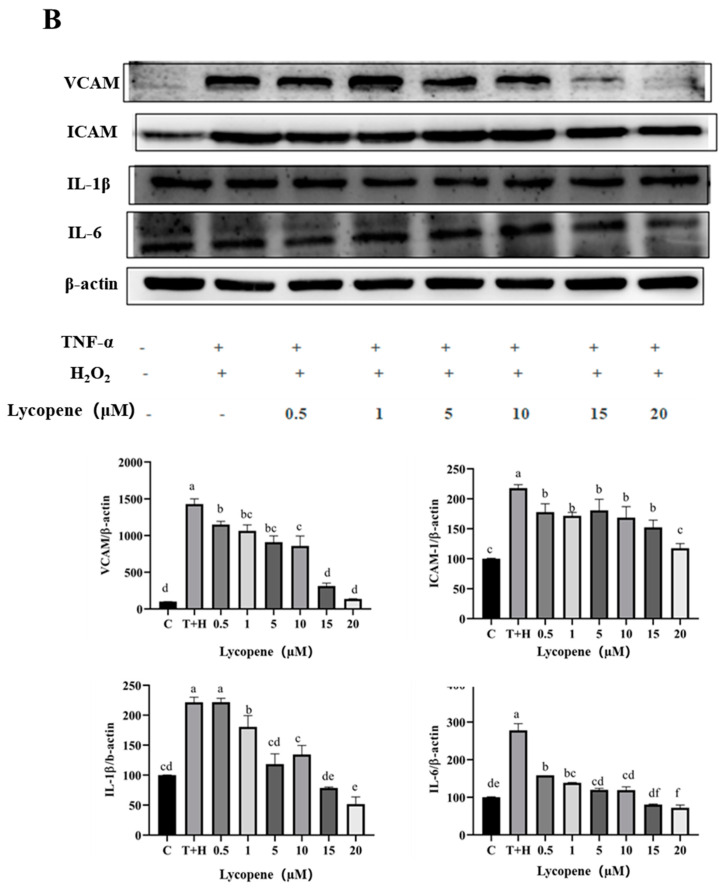

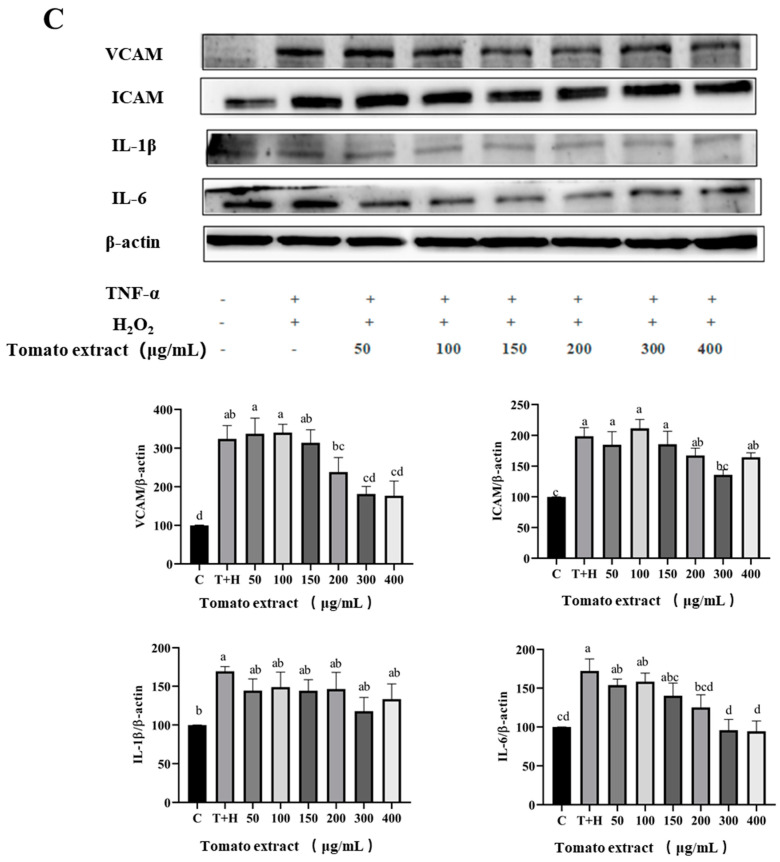
Expression of VCAM, ICAM, IL-1β, and IL-6 pro-inflammatory factor proteins induced by lutein, lycopene, and tomato extract in 200 μM H_2_O_2_ and 80 ng/mL TNF-α co-induced H9c2 cells. (**A**) Expression of VCAM, ICAM, IL-1β and IL-6 pro-inflammatory factor proteins induced by lutein. (**B**) Expression of VCAM, ICAM, IL-1β and IL-6 pro-inflammatory factor proteins induced by lycopene. (**C**) Expression of VCAM, ICAM, IL-1β and IL-6 pro-inflammatory factor proteins induced by tomato extract. Different letters (a, b, c, d, e, f) indicate significant differences between groups (*p* < 0.05).

The grayscale values of intracellular VCAM, ICAM, IL-1β, and IL-6 protein expressions in the control group were 100, and the T + H group (80 ng/mL TNF-α + 200 µM H_2_O_2_) significantly upregulated the inflammatory VCAM (2294), ICAM (222.89), IL-1β (151.3), and IL-6 (165.70). The expressions of inflammatory proteins were successfully induced. Compared with the T+H group, the expressions of VCAM, ICAM, IL-1β, and IL-6 protein decreased gradually with the increase in lycopene concentration (*p* < 0.05). Tomato extract decreased the expression of VCAM and IL-6 proteins in a dose-dependent manner (Figure 5). Moreover, 0.5–20 µM lutein, 0.5–20 µM lycopene, and 50–400 µg/mL tomato extract somewhat dose dependently reduced the 80 ng/mL TNF-α + 200 µM H_2_O_2_-induced upregulation of VCAM, ICAM, IL-1β, and IL-6 inflammatory factor proteins.

### 3.6. Effects of Lutein, Lycopene, and Tomato Extract on the Expression of Anti-Inflammatory Cytokines in H9c2 Cells

**Figure 6:**
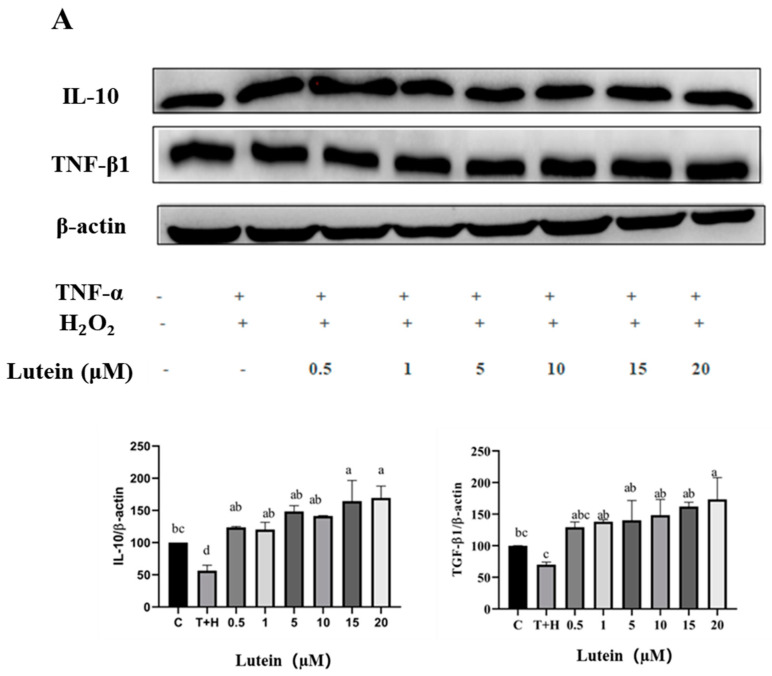

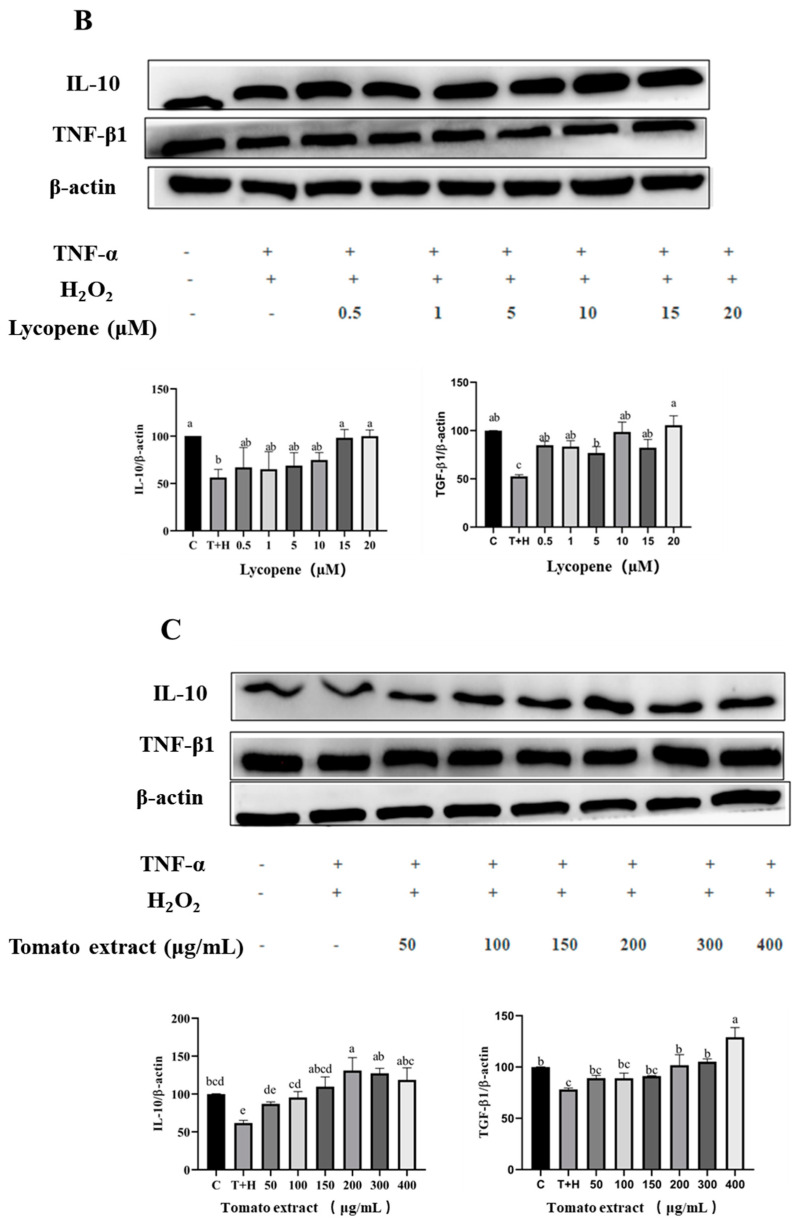
Expression of IL-10 and TGF-β1 anti-inflammatory factor proteins induced by lutein, lycopene, and tomato extract in 200 μM H_2_O_2_ and 80 ng/mL TNF-α co-induced H9c2 cells. (**A**) Expression of IL-10 and TGF-β1 anti-inflammatory factor proteins induced by lutein. (**B**) Expression of IL-10 and TGF-β1 anti-inflammatory factor proteins induced by lycopene. (**C**) Expression of IL-10 and TGF-β1 anti-inflammatory factor proteins induced by tomato extract. Different letters (a, b, c, d, e) indicate significant differences between groups (*p* < 0.05).

The T + H group (80 ng/mL TNF-α + 200 µM H_2_O_2_) significantly downregulated the expression of TGF-β1 (52.61–78.63) and IL-10 (56.37) anti-inflammatory factor proteins compared to the control group (*p* < 0.05). Moreover, 0.5–20 µM lutein, 0.5–20 µM lycopene, and 50–400 µg/mL tomato extract alleviated the 80 ng/mL TNF-α and 200 µM H_2_O_2_ co-induced downregulation of anti-inflammatory factor proteins (TGF-β1 and IL-10) to some extent (*p* < 0.05). Secondly, it showed a significant dose-dependent effect on the upregulation of TGF-β1 and IL-10. For example, at a lutein concentration of 0.5 µM, the grayscale values of TGF-β1 and IL-10 proteins expression were 129.31 ± 8.73 and 123.68 ± 1.78, respectively. When the concentration of lutein increased to 20 µM, the TGF-β1 and IL-10 proteins expression increased to 173.54 ± 34.2 and 169.48 ± 18.29, respectively (Figure 6).

### 3.7. Effects of Lutein, Lycopene, and Tomato Extract on iNOS in H9c2 Cells

**Figure 7:**
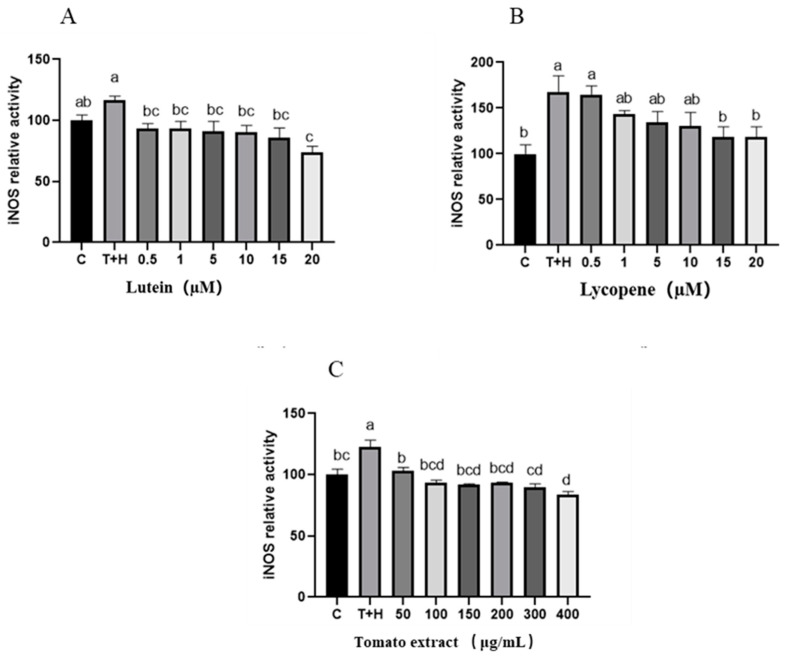
iNOS levels induced by lutein, lycopene, and tomato extract in 200 μM H_2_O_2_ and 80 ng/mL TNF-α co-induced H9c2 cells. (**A**) iNOS levels of H9c2 cells treated with varying concentrations of lutein. (**B**) iNOS levels of H9c2 cells treated with varying concentrations of lycopene. (**C**) iNOS levels of H9c2 cells treated with varying concentrations of tomato extract. Different letters (a, b, c, d) indicate significant differences between groups (*p* < 0.05).

Nitric oxide synthases, which include neurons (nNOS) and iNOS [35], act as vital enzymes in the formation of NO. Inflammatory disorders continue to develop and manifest as a result of excessive NO. As a result, the iNOS in H9c2 cells were detected following multiple treatments with varied dosages of lutein, lycopene, and tomato extract. Compared with the T + H group (80 ng/mL TNF-α + 200 µM H_2_O_2_), intracellular iNOS expression gradually decreased with increasing concentrations of lutein, lycopene, and tomato extract (*p* < 0.05), indicating that 0.5–20 μM lutein, 0.5–20 μM lycopene, and 50–400 μg/mL tomato extract inhibited the expression of iNOS and thus reduced NO production (Figure 7).

### 3.8. Activation of Nrf2 Pathway by Lutein, Lycopene, and Tomato Extract

The T + H (80 ng/mL TNF-α + 200 µM H_2_O_2_) group significantly inhibited Nrf2 (43.24 ± 3.07), NQO-1 (58.76 ± 6.96), and HO-1 (53.63 ± 1.06) protein expressions compared with the control group (*p* < 0.05). In addition, 0.5–20 µM lutein, 0.5–20 µM lycopene, and 50–400 µg/mL tomato extract pretreatment increased the expression of the Nrf2 signaling pathway downstream proteins (Nrf2, HO-1, and NQO-1) compared to the T + H group (*p* < 0.05), suggesting that 0.5–20 µM lutein, 0.5–20 µM lycopene, and 50–400 µg/mL tomato extract activated the Nrf2 signaling pathway.

**Figure 8:**
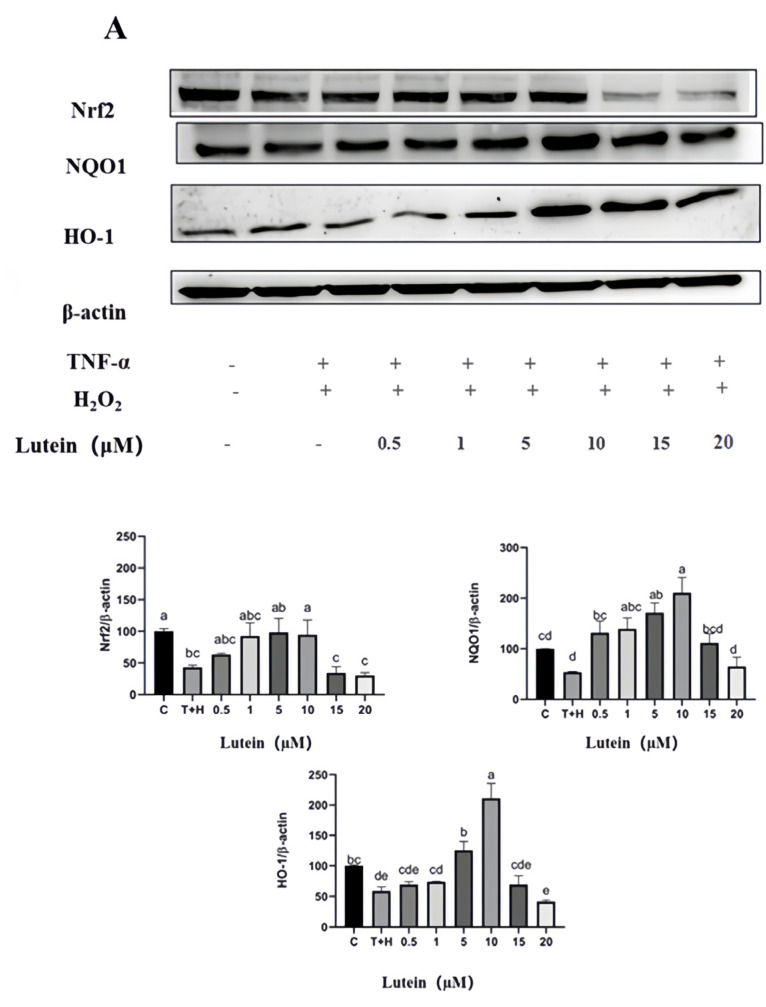

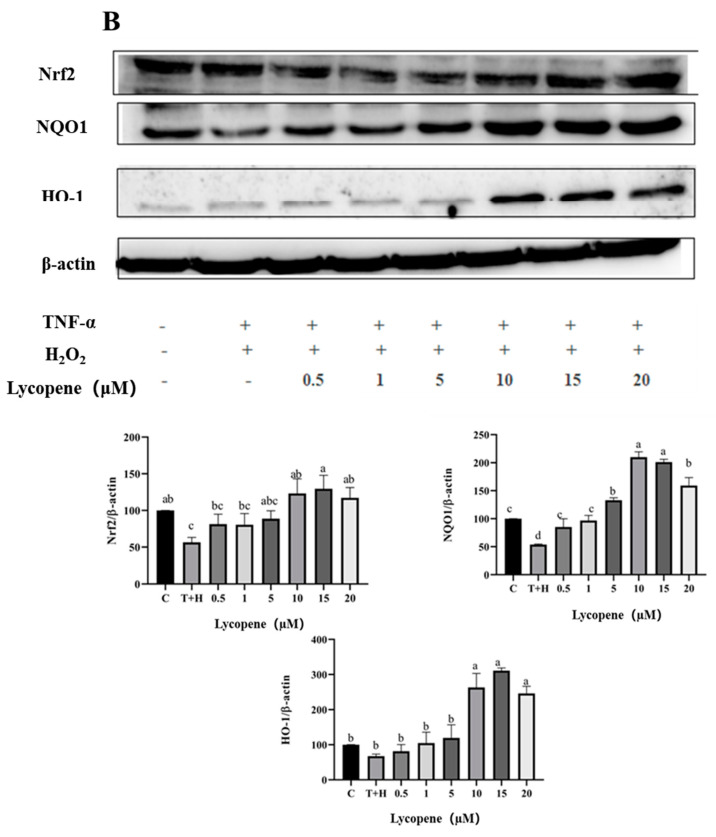

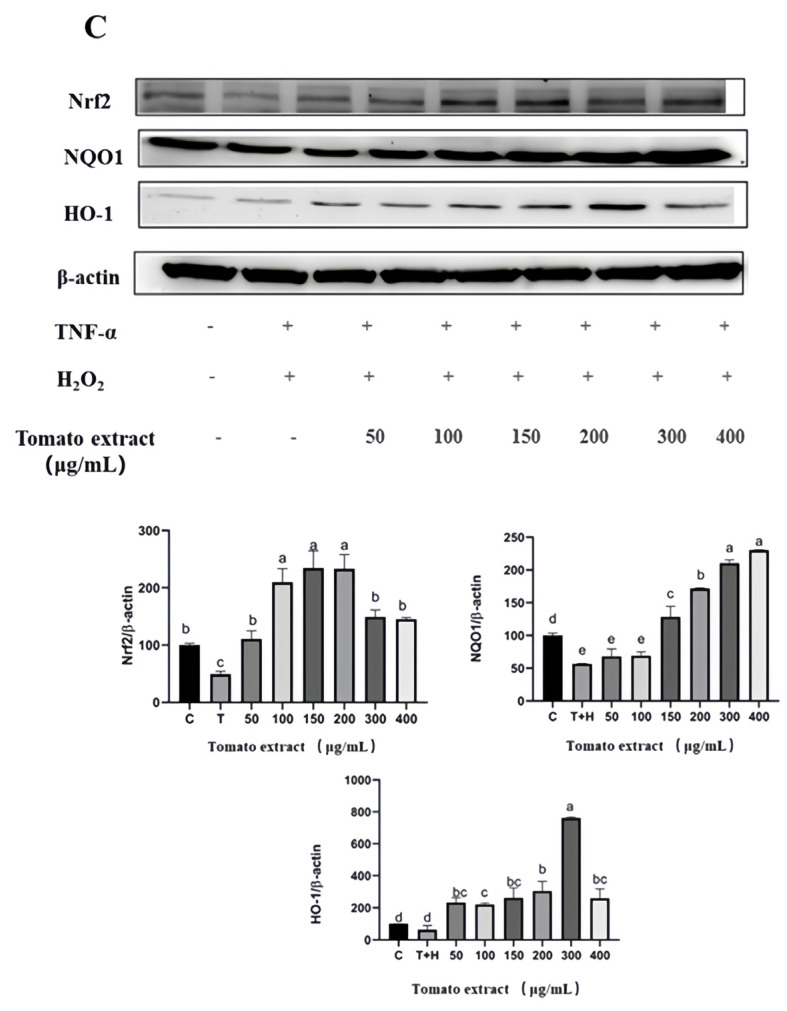
Nrf2, NQO-1 and HO-1 expression induced by lutein, lycopene, and tomato extract in 200 μM H_2_O_2_ and 80 ng/mL TNF-α co-induced H9c2 cells. (**A**) Nrf2, NQO-1 and HO-1 expression of H9c2 cells treated with varying concentrations of lutein. (**B**) NQO-1 and HO-1 expression of H9c2 cells treated with varying concentrations of lycopene. (**C**) NQO-1 and HO-1 expression of H9c2 cells treated with varying concentrations of tomato extract. Different letters (a, b, c, d, e) indicate significant differences between groups (*p* < 0.05).

On the other hand, the trends that emerged following various drug treatments were varied. From the results, 0.5–10 µM lutein and 0.5–10 µM lycopene dose dependently enhanced the expression of Nrf2, HO-1, and NQO-1 (*p* < 0.05), whereas 10–20 µM lutein and 10–20 µM lycopene dose dependently decreased the expression of Nrf2, HO-1, and NQO-1 (*p* < 0.05). The expression of antioxidant proteins downstream of the Nrf2 signaling pathway showed an increasing trend followed by a decreasing trend. For example, when the concentration of lutein was 0.5 µM, the expression of HO-1 was 69.2 ± 4.9. When the concentration of lutein increased to 10 µM, the expression of HO-1 increased to 211.2 ± 24.5. However, when the concentration of lutein continued to increase to 20 µM, the expression of HO-1 decreased to 41.52 ± 2.7 (Figure 8A).

The expression of antioxidant proteins downstream of the Nrf2 signaling pathway (Nrf2, NQO-1, and HO-1) was activated by 0.5–20 µM lutein. Moreover, the activation trend of 0.5–20 µM lycopene and 50–400 µg/mL tomato extract showed a trend of increasing and then decreasing. This is generally consistent with the changes in intracellular ROS induced by 0.5–20 µM lutein, 0.5–20 µM lycopene, and 50–400 µg/mL tomato extract. The modulation of the Nrf2 signaling pathway by different concentrations of lutein, lycopene, and tomato extract was likely to be the mechanism by which they produced low concentrations of antioxidant effects and high concentrations of pro-oxidant effects.

### 3.9. Lutein, Lycopene and Tomato Extract Promote Nrf2 Nuclear Transport

**Figure 9:**
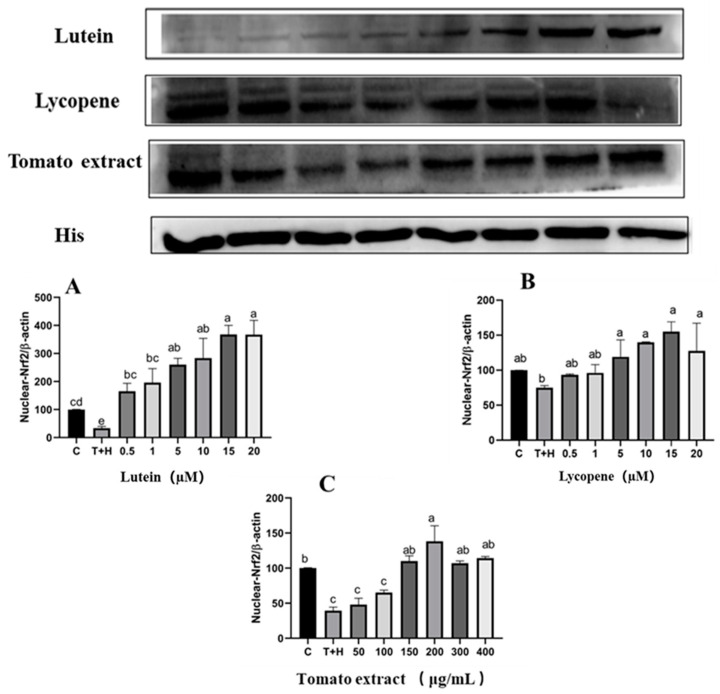
Nrf2 nuclear transport levels induced by lutein, lycopene, and tomato extract in 200 μM H_2_O_2_ and 80 ng/mL TNF-α co-induced H9c2 cells. (**A**) Nrf2 nuclear transport levels of H9c2 cells treated with varying concentrations of lutein. (**B**) Nrf2 nuclear transport levels of H9c2 cells treated with varying concentrations of lycopene. (**C**) Nrf2 nuclear transport levels of H9c2 cells treated with varying concentrations of tomato extract. Different letters (a, b, c, d, e) indicate significant differences between groups (*p* < 0.05).

The modifications of varying doses of lutein, lycopene, and tomato extract on Nrf2 nuclear transport in cells were identified utilizing Western blot. Simultaneously, 0.5–10 M lutein, 0.5–10 M lycopene, and 50–200 g/mL tomato extract increased the expression of Nrf2 protein in the nucleus (*p* < 0.05). When the concentration continued to increase, the expression of Nrf2 protein in the nucleus of 10–20 μM lutein, 10–20 μM lycopene, and 200–400 μg/mL tomato extract did not continue to increase (Figure 9). This is consistent with the expression results of downstream proteins of the Nrf2 signaling pathway. Lutein, lycopene, and tomato extract may act by facilitating the transfer of Nrf2 from the cytoplasm to the nucleus to activate the Nrf2 pathway, thereby activating downstream protein expression.

### 3.10. Effects of Lutein, Lycopene, and Tomato Extract on NF-κB Activation

**Figure 10:**
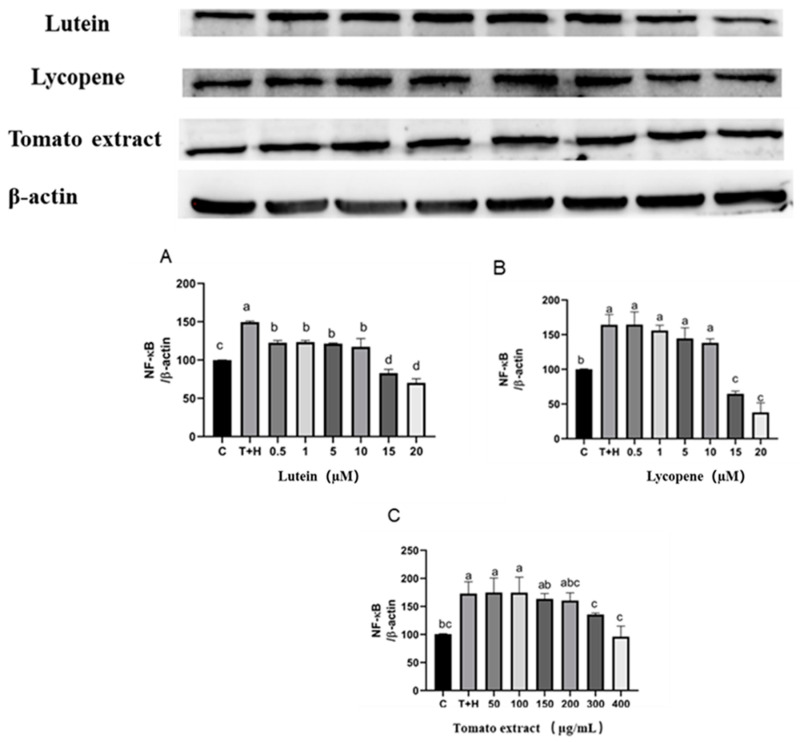
NF-κB p-p65 protein expression induced by lutein, lycopene, and tomato extract in 200 μM H_2_O_2_ and 80 ng/mL TNF-α co-induced H9c2 cells. (**A**) NF-κB p-p65 protein expression of H9c2 cells treated with varying concentrations of lutein. (**B**) NF-κB p-p65 protein expression of H9c2 cells treated with varying concentrations of lycopene. (**C**) NF-κB p-p65 protein expression of H9c2 cells treated with varying concentrations of tomato extract. Different letters (a, b, c, d) indicate significant differences between groups (*p* < 0.05).

Activation of the NF-κB pathway upregulates the production of pro-inflammatory cytokines, while changes in p-p65 expression can determine whether the NF-κB pathway was activated [36]. The expression of NF-κB p-p65 in the T + H group (80 ng/mL TNF-α + 200 µM H_2_O_2_) was significantly higher compared with that in the control group (164.3 ± 14.85), indicating that 80 ng/mL TNF-α + 200 µM H_2_O_2_ can effectively activate the NF-κB pathway. Lutein, lycopene, and tomato extract pretreatment can inhibit the expression of NF-κB p-p65 to different degrees compared to the T + H group. When the concentration of lutein increased from 10 µM to 20 µM, the grayscale value of NF-κB p-p65 protein decreased significantly from 164.5 ± 18.27 to 37.97 ± 17.35 (*p* < 0.05). At the same time, lycopene and tomato extract also decreased the expression of NF-κB p-p65 in a dose-dependent manner, indicating that the pathway was inhibited (Figure 10).

### 3.11. Effects of Different Concentrations of Lutein, Lycopene, and Tomato Extract on NF-κB Nuclear Transport

**Figure 11:**
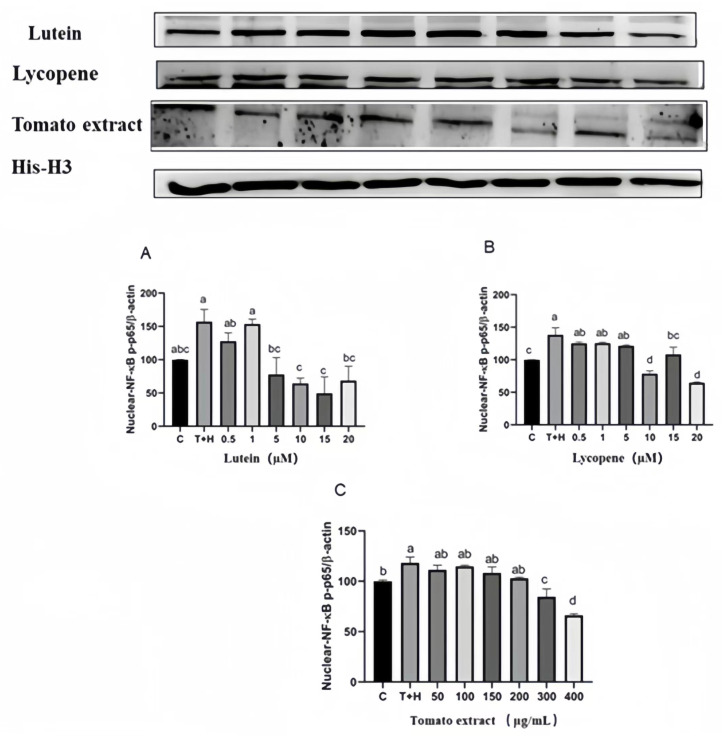
NF-κB p65 nuclear transport levels induced by lutein, lycopene, and tomato extract in 200 μM H_2_O_2_ and 80 ng/mL TNF-α co-induced H9c2 cells. (**A**) NF-κB p65 nuclear transport levels of H9c2 cells treated with varying concentrations of lutein. (**B**) NF-κB p65 nuclear transport levels of H9c2 cells treated with varying concentrations of lycopene. (**C**) NF-κB p65 nuclear transport levels of H9c2 cells treated with varying concentrations of tomato extract. Different letters (a, b, c, d) indicate significant differences between groups (*p* < 0.05).

Compared to the control group, the expression of NF-κB in the nucleus of the T + H group (80 ng/mL TNF-α + 200 µM H_2_O_2_, 38.3 ± 10.96–157.1 ± 18.41) was significantly higher (*p* < 0.05). Furthermore, 0.5–20 µM lutein (127.7 ± 25.96–49.58 ± 24.63), 0.5–20 µM lycopene (125.3 ± 2.14–96.16 ± 18.79), and 50–400 µg/mL tomato extract (111.3 ± 4.63–66.15 ± 1.48) significantly decreased nuclear NF-κB expression (*p* < 0.05) (Figure 11). Quantities of 0.5–20 µM lutein, 0.5–20 µM lycopene, and 50–400 μg/mL tomato extract can inhibit the expression of nuclear NF-κB protein to varying degrees and thus exert anti-inflammatory effects.

### 3.12. TGF-β1/IL-10 Regulates the Effect of Nrf2 and NF-κB mRNA Expression

Inhibitory modifications to the NF-κB signaling pathway increased with concentration, although the capacity of 10–20 µM lutein, 10–20 µM lycopene, and 200–400 µg/mL tomato extract to activate Nrf2 signaling pathway and downstream protein expression reduced with the increase in concentrations. As a result, it was speculated that increased production of anti-inflammatory molecules (TGF-1 and IL-10) suppressed the expression of Nrf2 signaling pathway-related proteins. In this work, siRNA gene-silencing technology was utilized to silence anti-inflammatory factors TGF-1 and IL-10, and then Q-PCR technology was employed to determine the expression of Nrf2, HO-1, and NF-κB genes.

#### 3.12.1. Regulation of Nrf2 mRNA, HO-1 mRNA, and NF-κB mRNA by TGF-β1

**Figure 12:**
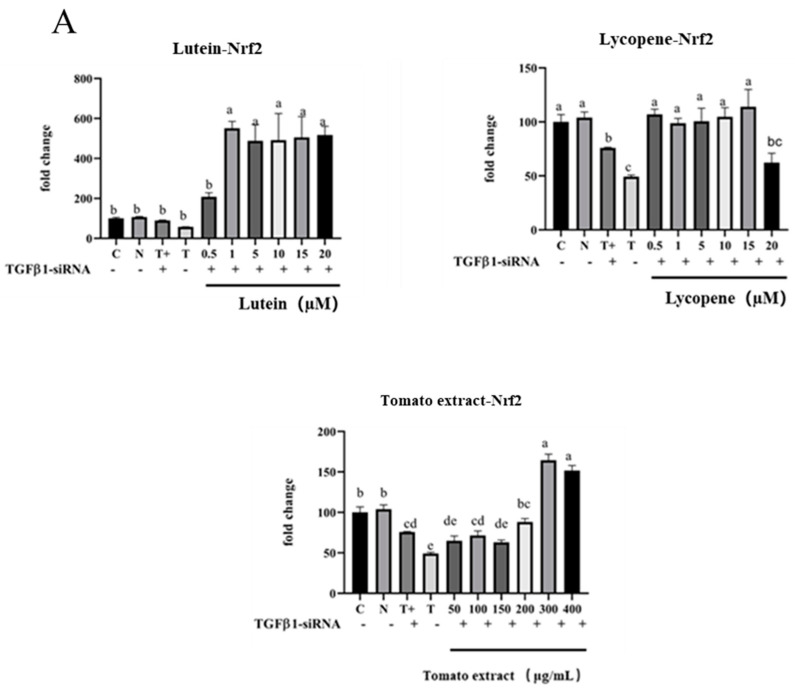

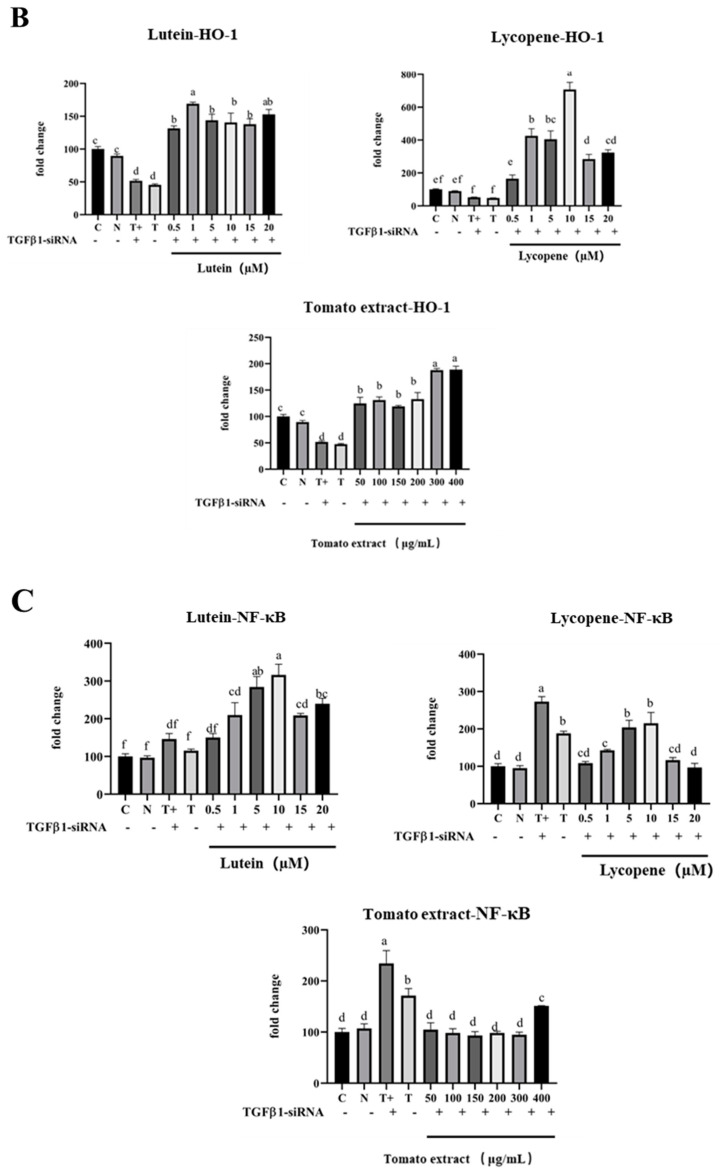
Effect of silencing TGF-β1 on Nfr2 mRNA, HO-1 mRNA and NF-κB mRNA. (**A**) Effect of silencing TGF-β1 on Nrf2 mRNA after pretreatments. (**B**) Effect of silencing TGF-β1 on HO-1 mRNA after pretreatments. (**C**) Effect of silencing TGF-β1 on NF-κB mRNA after pretreatments. C is control, NC is negative control, T + H is the model group of 80 ng/mL TNF-α + 200 µM H_2_O_2_ transfected with TGF-β1, and T is the model group of 80 ng/mL TNF-α + 200 µM H_2_O_2_ not transfected with TGF-β1. Different letters (a, b, c, d, e, f) indicate significant differences between groups (*p* < 0.05).

The expression of the Nrf2 gene in the TGF-β1 gene-silenced model group (75.97 ± 0.57%) was significantly higher than that in the non-silenced model group (49.28 ± 1.52%). The expression of Nrf2 was significantly higher (*p* < 0.05) after treatment with different concentrations of lutein, lycopene, and tomato extract under the premise of TGF-β1 gene silencing. Meanwhile, the expression of Nrf2 was no longer decreased by high concentrations of lutein (10–20 µM) lycopene (10–15 µM), or tomato extract (200–400 µg/mL) pretreatment. This indicates that Nrf2 may depend on TGF-β1 to function. The Nrf2 expression level was downregulated by TGF-β1 at high concentrations of lutein, lycopene, and tomato extract (Figure 12), thus exerting a pro-oxidant effect.

Lutein, lycopene, and tomato extract may be controlled by TGF-β1 to affect HO-1 expression and exert antioxidant effects. After the treatment with different concentrations of lutein and lycopene, HO-1 mRNA expression showed a trend of increasing low concentration and decreasing high concentration compared to the T + group (T + was a model group transfected with TGF-β1 at 80 ng/mL TNF-α + 200 µM H_2_O_2_, Figure 12B). For example, HO-1 mRNA expression increased from 131.7 ± 3.79% to 169.1 ± 2.05% after 0.5–1 µM lutein pretreatment. The mRNA expression of HO-1 in 0.5–10 µM tomato extract was increased from 124.6 ± 11.71% to 188.8 ± 6.54% (*p* < 0.05), and HO-1 mRNA expression in high concentrations of lutein (10–20 µM) and tomato extract (200–400 µg/mL) no longer decreased. These results suggested that HO-1 may be dependent on TGF-β1 to exert antioxidant effects.

Lutein may depend on TGF-β1 downregulation of NF-κB mRNA expression to exert the anti-inflammatory effects. After 0.5–20 µM lutein pretreatment, the expression of NF-κB mRNA was higher (150.3 ± 10.58%–316.1 ± 28.54%) than that in the T + group (157.1 ± 14.85%) and significantly increased (*p* < 0.05). The results indicate that the expression of NF-κB was increased after inhibition of TGF-β1, and the NF-κB signaling pathway was dependent on TGF-β1 to play an anti-inflammatory role. However, compared with the T + group, NF-κB mRNA expression after different concentrations of lycopene and tomato extract was not increased (Figure 12), indicating that lycopene and tomato extract may play anti-inflammatory effects on NF-κB downregulated by other anti-inflammatory factors.

#### 3.12.2. Regulation of Nrf2 mRNA, HO-1 mRNA, and NF-κB mRNA by IL-10

**Figure 13:**
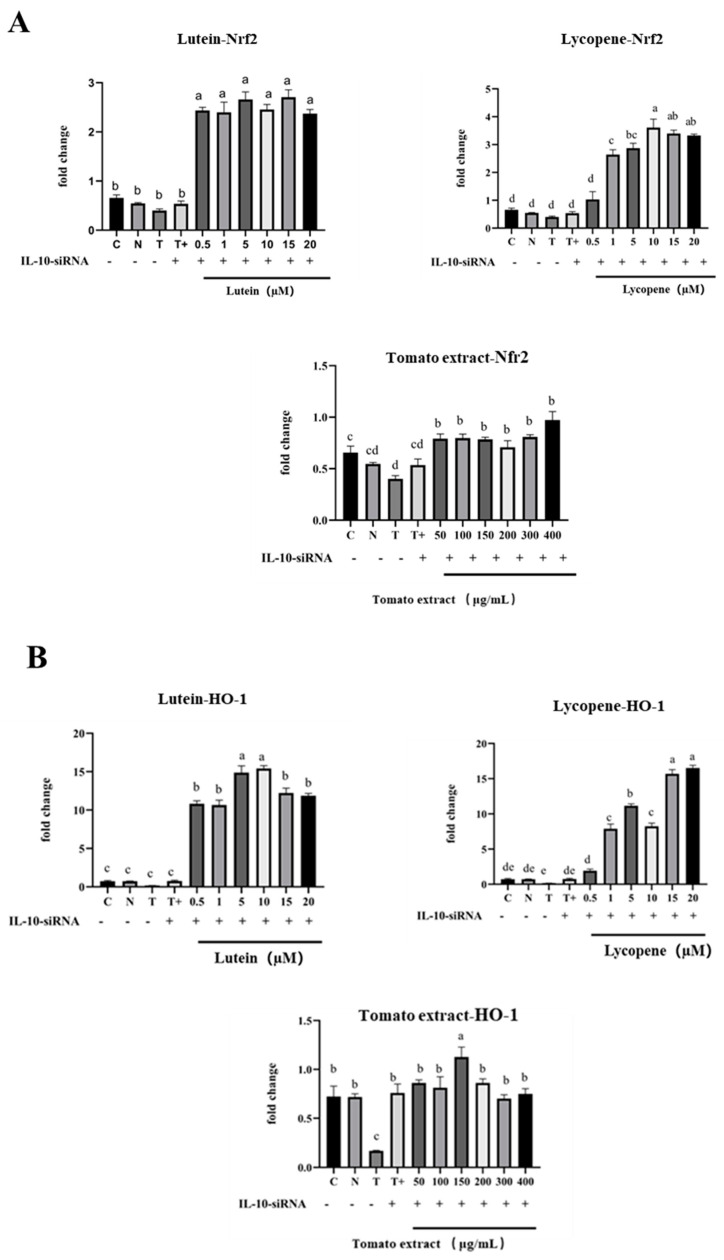

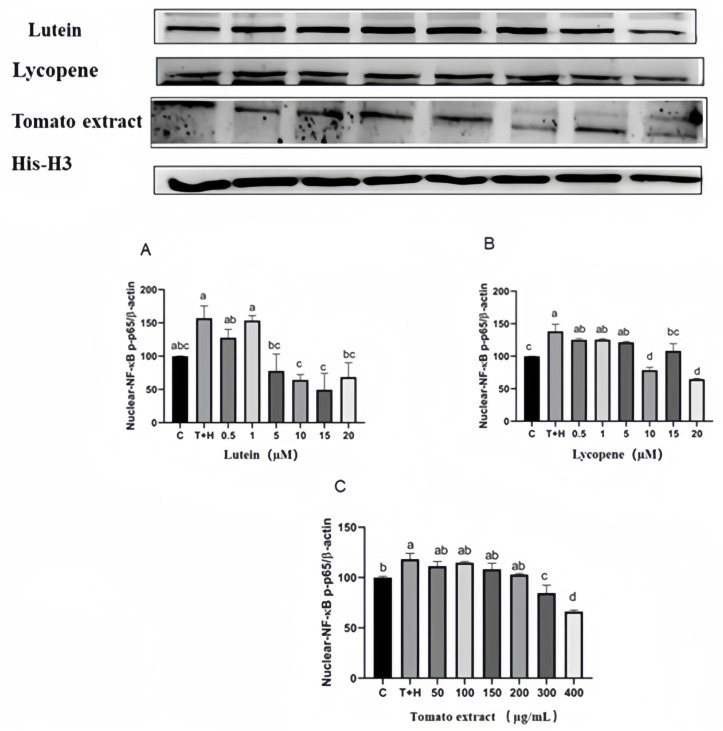
Effect of silencing IL-10 on Nfr2 mRNA, HO-1 mRNA, and NF-κB mRNA. (**A**) Effect of silencing IL-10 on Nfr2 mRNA after pretreatments. (**B**) Effect of silencing IL-10 on HO-1 mRNA after pretreatments. (**C**) Effect of silencing IL-10 on NF-κB mRNA after pretreatments. C is Control, NC is Negative Control, T + is the model group of 80 ng/mL TNF-α + 200 µM H_2_O_2_ transfected with IL-10, and T is the model group of 80 ng/mL TNF-α + 200 µM H_2_O_2_ not transfected with IL-10. Different letters (a, b, c, d, e) indicate significant differences between groups (*p* < 0.05).

High concentrations of lutein, lycopene, and tomato extract may depend on IL-10 while downregulating Nrf2 expression to exert a pro-oxidant effect. Under the premise of silencing the IL-10 gene, the expression of Nrf2 was significantly increased after treatment with different concentrations of lutein, lycopene, and tomato extract (*p* < 0.05). In addition, the expression of Nrf2 no longer decreased at high concentrations of lutein (10–20 µM), lycopene (10–15 µM), and tomato extract (200–400 µg/mL) (Figure 13A).

Lutein, lycopene, and tomato extract may be controlled by IL-10 to affect the expression of HO-1. High concentrations of lutein, lycopene, and tomato extract may downregulate the HO-1 expression depending on IL-10, thus playing a role in promoting oxidation. Under the premise of silencing the IL-10 gene, HO-1 expression was significantly increased after the treatment with different concentrations of lutein, lycopene, and tomato extract (*p* < 0.05). In addition, the expression of HO-1 did not decrease after high concentration of lycopene (10–15 µM), indicating that HO-1 may depend on IL-10 to act.

Lutein, lycopene, and tomato extract may be controlled by IL-10, which may affect the expression of NF-κB and play an anti-inflammatory role. Under the premise of silencing the IL-10 gene, the expression of NF-κB was significantly increased after different concentrations of lutein, lycopene, and tomato extract (*p* < 0.05). In addition, the expression of NF-κB in high concentrations of lutein (10–20 µM), lycopene (10–15 µM), and tomato extract (200–400 µg/mL) no longer decreased, indicating that Nrf2 may depend on IL-10. High concentrations of lutein, lycopene, and tomato extract may depend on IL-10 to downregulate the expression of NF-κB, thus playing an anti-inflammatory role.

## 4. Discussion

### 4.1. The Explanation of Antioxidation and Pro-Oxidation Effects

The DCFH-DA assay has been widely applied in cell studies and used as a marker of cellular oxidative stress. The oxidation of DCFH produces DCF, a fluorescent compound that was originally thought to be useful as a specific indicator of H_2_O_2_. However, it has been shown that DCFH is oxidized by other ROS [37]. In this study, it was more meaningful to detect the total ROS content in the process of cellular oxidative stress because it also involves the production of ROS in the process of chronic inflammation. The oxidative damage that the T + H group (200 µM H_2_O_2_ + 80 ng/mL TNF-α) caused in H9c2 cells was altered by 0.5–20 µM lutein, 0.5–20 µM lycopene, and 50–400 µg/mL tomato extract. It was discovered that the antioxidant effects of 0.5–5 μM lutein, 0.5–5 μM lycopene, and 50–200 μg/mL tomato extract were continually strengthened. The antioxidant activity of carotenoids was dramatically impacted by the chemical structure, membrane binding site, concentration, and oxygen partial pressure [38]. All C=C bonds exist in the straight-chain structure of lycopene in coplanar form, and unpaired electrons created at the allyl C-4 location are prone to delocalization [39]. Accordingly, these structural characteristics determine that lycopene has a potent ability to scavenge free radicals and block singlet oxygen. Lutein plays an antioxidant role by scavenging oxygen free radicals and hydroxyl free radicals and inhibiting lipid peroxidation [40]. Due to its distinctive chemical composition, particularly the conjugated double bonds and the two hydroxyl groups at the end of the chain side, lutein is resistant to oxidation [41]. The conjugated double bond structure of the main carotenoids of tomatoes enables carotenoids to effectively clear ROS and RNS produced by pathophysiological processes or normal metabolic processes [42].

The total glutathione peroxidase assay is a simple UV kit used to detect glutathione peroxidase activity in cells. The organic peroxide reagent provided in this kit is cumene hydroperoxide (Cum-OOH). The organic peroxide reagent (Cum-OOH) provided by the GPX Assay Kit does not react with glutathione in the presence of glutathione peroxidase and is not catalyzed by intracellular catalase. Therefore, the activity of glutathione peroxidase can be more specifically detected. The capacity of the main carotenoids of tomatoes to scavenge free radicals reduced when the concentration of lutein was more than 5 µM, that of lycopene was more than 5 µM, and that of tomato extract was more than 200 ng/mL. Moreover, the activity of antioxidant enzymes such as SOD and GPX ceased to rise and even fell. These carotenoids showed pro-oxidative action at high dosages despite having antioxidant activity at low amounts. Previous research indicated that the pro-oxidation effect of carotenoids may be due to the imbalance in the cellular redox state, an excessive concentration of carotenoids, or an excessively high oxygen partial pressure [43]. Carotenoids are antioxidants that slow down or stop the chain of free radical reactions when oxygen saturation is low [44]. Nevertheless, when the oxygen concentration was high, carotenoid radicals were vulnerable to automatic oxidation by reacting with molecular oxygen while becoming aggressive carotenoid peroxy radicals, which promoted the lipid peroxidation and the oxidative damage of other biomolecules (such as DNA and proteins) [45]. When carotenoids are absorbed in large quantities, carotenoids can reduce antioxidant capacity and potentially trigger pro-oxidation processes [46]. When carotenoids levels are high, they can combine with ROS to form oxidative compounds that may have a pre-carcinogenic impact and cause harm to the organs and tissues. β-carotene oxidative metabolites have been proven in studies to promote the damage of benzopyrene to DNA [47]. Carotenoids in high quantities can also change the characteristics of cell membranes and enhance their permeability. According to research on the association between DNA damage and cell membrane permeability, carotenoids may enhance the permeability of water-soluble ROS, increasing the risk of DNA damage [48]. High concentrations of carotenoids may assemble or crystallize in cells, causing major changes in biochemical features such as membrane fluidity and permeability, which ultimately lead to pro-oxidation. The explanations given above for carotenoids at high concentrations inducing cell damage or pro-oxidation also explain why, in this investigation, lutein, lycopene, and tomato extract at elevated concentrations displayed pro-oxidation features.

### 4.2. Interpretation of Anti-Inflammatory Vitality

In this research work, lutein, lycopene, and tomato extract enhanced the anti-inflammatory effects in a dose-dependent manner. The anti-inflammatory effects of lutein (0.5–20 μM), lycopene (0.5–20 μM), and tomato extract (50–400 μg/mL) were simultaneously strengthened with higher concentrations. Previous studies have also verified that the anti-inflammatory effect of lycopene is dose dependent. Lycopene at doses of 20 mg/kg and 60 mg/kg could dramatically lower the content of inflammatory factors in serum and boost non-alcoholic fatty liver caused by high intakes of fat and sugar [24]. The regulating effect of lycopene at 60 mg/kg was also discovered to be substantially superior to that at 20 mg/kg. These are similar to our study results that showed lutein, lycopene, and tomato extract enhance anti-inflammatory effects in a dose-dependent manner.

### 4.3. Activation of Nrf2 Signaling Pathway and Inhibition of NF-κB Signaling Pathway

Nrf2 is a key transcription factor when cells are under oxidative stress and plays an important role in maintaining cell homeostasis [49]. To preserve the cell stability under normal conditions, Nrf2 interacts with Keap1 in the cytoplasm [50]. Instead, in stressful circumstances, Nrf2 separates from the Keap1 protein and transfers into the nucleus, triggering the transcription of a variety of antioxidant genes, including GCLC, HO-1, and NQO1 [51,52]. Treatments of 0.5–20 μM lutein, 0.5–20 μM lycopene, and 50–400 μg/mL tomato extract can stimulate the transfer of Nrf2 to the nucleus. Nrf2 also binds antioxidant response elements to increase the production of cellular antioxidant defense-related proteins, which alleviate oxidative stress caused by 80 ng/mL TNF-α + 200 µM H_2_O_2_. This proved that lutein, lycopene, and tomato extract at low concentrations activated the Nrf2 signaling pathway by enhancing Nrf2 nuclear transport. Nevertheless, as lutein, lycopene, and tomato extract concentrations increased, the expression of Nrf2 in the nucleus either stopped growing or showed a trend of decreasing after saturation, which was consistent with the trend of the intracellular ROS level and SOD and GPX antioxidant enzyme activity. These findings confirm that the pro-oxidation of high concentrations of lutein lycopene and tomato extracts are directly related to the Nrf2 signaling pathway. This may be the primary mechanism that exerts antioxidation at low concentrations and pro-oxidation at high concentrations.

Lutein, lycopene, and tomato extract have potent anti-inflammatory properties, decreasing the expression of pro-inflammatory factors in H9c2 cells (VCAM, ICAM, IL-1, and IL-6), while increasing the expression of anti-inflammatory factors (TGF-1 and IL-10), and decreasing the activity of the iNOS enzyme may be crucial to the anti-inflammatory activities. In this research work, lutein, lycopene, and tomato extract enhanced the anti-inflammatory effects in a dose-dependent manner. The anti-inflammatory effects of lutein (0.5–20 μM), lycopene (0.5–20 μM), and tomato extract (50–400 μg/mL) were simultaneously strengthened with higher concentrations. This was mainly because IκBα was induced to undergo phosphorylation after stimulation by TNF-α. Phosphorylated IκBα degrades rapidly, releasing NF-κB into the nucleus and controlling transcriptional genes [53]. Lutein, lycopene, and tomato extract can suppress the activation of the NF-κB signaling pathway by inhibiting NF-κB p65 nuclear migration in a dose-dependent manner. Our results demonstrated that lutein, lycopene, and tomato extract possess potent anti-inflammatory effects via inhibiting the NF-κB signaling pathway.

### 4.4. Modulation of Nrf2 and NF-κB Signaling Pathways by Anti-Inflammatory Factors

Oxidative stress and inflammation are controlled through functional interactions and cross-talk between Nrf2 and NF-κB pathways to maintain a sustainable balance of cellular redox states [54]. Additionally, intricate molecular interactions that work through both transcriptional and post-transcriptional mechanisms are responsible for the interaction between Nrf2 and NF-κB pathways [55]. Although numerous previous studies have produced compelling evidence for interactions of Nrf2 and NF-κB signaling pathways, the complex conditions and dynamics between these cross-talk variations remain unclear.

The NF-κB signaling pathway was consistently blocked by treatments with lutein (10–20 µM), lycopene (10–20 µM), and tomato extract (200–400 µg/mL), and the results showed a decreasing trend in the expression of the downstream protein of the Nrf2 signaling pathway in H9c2 cells, as well as the accumulation of Nrf2 in the nucleus. As a result, it was hypothesized that increased production of anti-inflammatory factors would suppress the expression of Nrf2 signaling pathway-related proteins. TGF-β1 and IL-10, representative inhibitory cytokines, play crucial roles in the regulation of immune homeostasis and have been shown to have outstanding therapeutic potential in inflammatory diseases. Hence, it was conjectured that the increase in anti-inflammatory factor (TGF-β1 and IL-10) may be responsible for the decline in the downstream protein and nuclear transport of the Nrf2 pathway. The expressions of TGF-β1 and IL-10 mRNA were silenced via the siRNA gene-silencing technique to investigate the regulation of anti-inflammatory factors TGF-β1 and IL-10 on the Nrf2 signaling pathway and NF-κB signaling pathway. 

Under the premise of silencing the TGF-β1 gene, the expression of Nrf2 was significantly increased after treatment with different concentrations of lutein lycopene and tomato extract (*p* < 0.05). Meanwhile, lutein lycopene and tomato extract may be controlled by TGF-β1 to affect the expression of HO-1 and play an antioxidant role. After inhibiting the expression of TGF-β1 gene, the expression of NF-κB increased, and the NF-κB signaling pathway may depend on TGF-β1 for its anti-inflammatory effects.

Similarly, after silencing the IL-10 gene, the expression of Nrf2 increased considerably following treatment with lutein lycopene and tomato extract at different dosages (*p* < 0.05). High levels of lutein, lycopene, and tomato extract may rely on IL-10 to suppress the production of HO-1, thus increasing oxidation. The expression of HO-1 was considerably raised following treatment with lutein, lycopene, and tomato extract at different dosages, whereas the IL-10 gene was silenced (*p* < 0.05). The expression of NF-κB was significantly increased after treatment with lutein, lycopene, and tomato extract under the condition of silencing the IL-10 gene (*p* < 0.05), indicating that lutein, lycopene, and tomato extract have a significant role in anti-inflammatory activity through the expression of NF-κB by controlling IL-10.

According to the results, the silenced anti-inflammatory factor (TGF-β1 and IL-10) can upregulate the expression of Nrf2/HO-1 and NF-κB mRNA to a certain extent. High concentrations of lutein, lycopene, and tomato extract downregulate Nrf2/HO-1 and NF-κB signaling pathways depending on TGF-β1 and IL-10, thus exerting low-concentration antioxidant and anti-inflammatory effects. Antioxidant and anti-inflammatory effects are effective therapeutic targets for CVD, and targeting TGF-β1 and IL-10 to regulate Nrf2/HO-1 and NF-κB signaling pathways may be a new therapeutic strategy for CVD. This finding will assist in properly elucidating the underlying molecular mechanisms of antioxidants at low concentrations and anti-inflammatory effects at high concentrations, and more effectively guide the dose–response relationship between lutein, lycopene, and tomato extract in preventing the occurrence of CVD.

## 5. Conclusions

It was found that lutein, lycopene, and tomato extract primarily exhibited antioxidant effects at low concentrations and pro-oxidation effects at high concentrations. In addition, the higher the concentration of lutein, lycopene, and tomato extract, the greater the anti-inflammatory activity. Lutein, lycopene, and tomato extract can exert antioxidant effects via activating the Nrf2 signaling pathway and exert anti-inflammatory effects by inhibiting the NF-κB signaling pathway.

Furthermore, the expression of Nrf2 downstream proteins (Nrf2, NQO1, HO-1) and nuclear translocation of Nrf2 were suppressed after treatment with high concentrations of lutein, lycopene, and tomato extract. This also unequivocally proved that the Nrf2 signaling pathway can function as both a low-concentration antioxidant and a high-concentration pro-oxidant, while the anti-inflammatory effect of high concentrations may occur via the inhibition of the nuclear shift of NF-κB. TGF-β1 and IL-10 can target the regulation of Nrf2/HO-1 and NF-κB to achieve a low concentration of antioxidants and a high concentration of anti-inflammatory effects. Nrf2 and NF-κB signaling pathways, and anti-inflammatory factors such as TGF-β1 and IL-10, may be potential therapeutic targets for CVD.

## Data Availability

The data presented in this study are available on request from the corresponding author.

## Supplementary Materials

The following supporting information can be downloaded at: https://www.mdpi.com/article/10.3390/nu15214652/s1, Table S1: Primer sequence of target gene; Figure S1: HPLC-VWD chromatogram at 450 nm of lutein (A), lycopene (B), and tomato extract (C); Figure S2: The viability effect of lutein, lycopene, and tomato extract on H9c2 cells.
