# Supplementary material for: The Antioxidant and Anti-Inflammatory Effects of the Main Carotenoids from Tomatoes via Nrf2 and NF-κB Signaling Pathways
Source: Nutrients. 2023 Nov 2;15(21):4652. doi: 10.3390/nu15214652 (PMC10650085; doi:10.3390/nu15214652)
Supplement: Supplementary file 1 [file nutrients-15-04652-s001.zip › nutrients-2663763-SI.pdf]

Supplementary Materials

Table S1. Primer sequence of target gene

Target gene	Primer sequence
Nrf2	Forward AAGCACAGCCAGCACATTCTCC
	Reverse TGACCAGGACTCACGGGAACTTC
HO-1	Forward TGGGTTCTGCTTGTTTCGCTCTATC
	Reverse CAGGTGTCCAGGGAAGGCTTTAAG
NF-κB	Forward TGTGGTGGAGGACTTGCTGAGG
	Reverse AGTGCTGCCTTGCTGTTCTTGAG
TGF-β1	Forward CTGGCACTGCTTCCCGAATGTC
	Reverse GACCGCAACAACGCAATCTATGAC
β-actin	Forward GCTGTGCTATGTTGCCCTAGACTTC
	Reverse GGAACCGCTCATTGCCGATAGTG

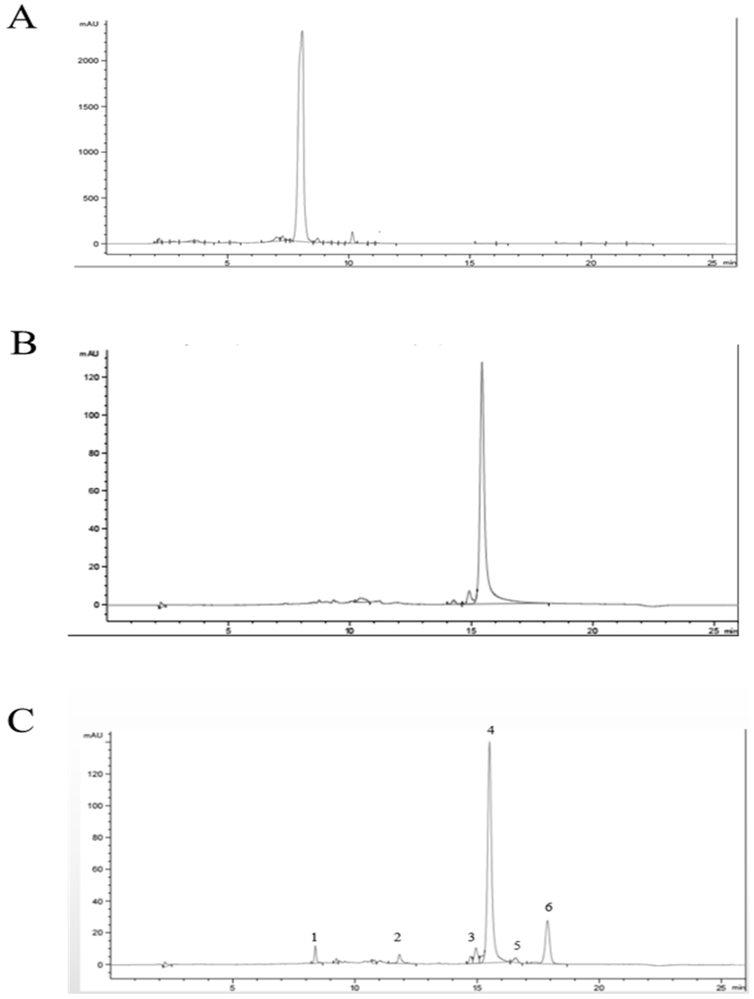

Figure S1. HPLC-VWD chromatogram at 450 nm of lutein (A), lycopene (B) and tomato extract (C).

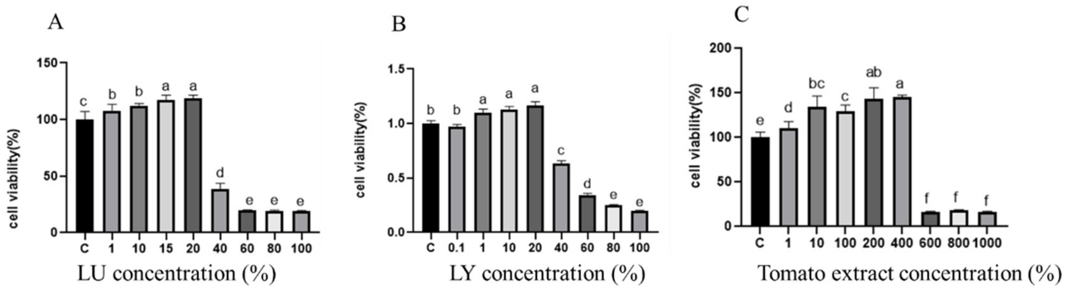

Figure S2. The viability effect of lutein, lycopene and tomato extract on H9c2 cells. A is the effect of different concentrations of lutein on H9c2 cell viability. B is the effect of different concentrations of lycopene on H9c2 cell viability. C is the effect of different concentrations of tomato extract on H9c2 cell viability. The horizontal coordinate C indicates the control group. Different letters (a, b, c, d, e, f) indicate significant differences between groups ($p < 0.05$).
